# Supplementary material for: Chorioamnionitis and its associated factors among women admitted to the maternity unit of Public Hospitals in Addis Ababa, Ethiopia
Source: PLOS Glob Public Health. 2025 Jul 14;5(7):e0004467. doi: 10.1371/journal.pgph.0004467 (PMC12258553; doi:10.1371/journal.pgph.0004467)
Supplement: Attachment — Submitted filename: Reviewer comment Chorio.docx [file pgph.0004467.s001.docx]

**Review report: Chorioamionitis and its associated factors among admitted women in maternity unit of Public Hospitals in Addis Ababa, Ethiopia.**

**Proposed title:** **Review report: Chorioamionitis and its associated factors among admitted women in maternity unit in selected Public Hospitals in Addis Ababa, Ethiopia.**

**Methods:**

Page 7: Line 89: The study was conducted from June 15 to July 15, 2024 G.C. Write the GC in full for avoiding confusion as its not a known world out of Ethiopia.

Page 8: Line 93:, **all clients** admitted to..: This word all clients must be changed to all pregnant women attended the maternity of ….

**Sampling technique and procedure:**

Explain how three hospitals were only selected. How many hospitals with maternity services in Adis Ababa? Was really randomly or purposefully selected? If it was randomly carried out, please elaborate on this, state all public hospitals and the procedure you used to select

Study Variables

Page 9: 126 Dependent Variable: Chorioamnionitis: explain how this variable was obtained. Was there any confirmation test done? Have you set the cutoff of signs and symptoms as you mentioned diagnosis of Chorioamnionitis on the line: 134 **Operational definition: Were all have to be fulfilled as citated: explain how you judged the overall results for Chorioamnionitis according to the definition.**

Page 9: line 130: Start by writing in full these words such as: PROM

**The data quality control:**

Page 10: Line 148, 149 …..pre-test was done on 5% : Just put the reference of the extent or percentage of sample size. Is it 5%?

Were other quality control measures (Validity and Reliability test) used?

**Page 10: line: 155. Data analysis**

Include the measurement of Chorioamnionitis, what the measures used for qualifying it: Yes or No?? or Cutoff?? Confirmation test?? Or other?

Why haven’t you used Chi-square or Fisher test instead of using the same test in bivariate and multivariate? For confounding adjustments.

Could you add a small paragraph of ethical clearance?

**Results:**

Page 11-16: 172 Table 1, table 2 and table 3. Separate the frequency and the percentage. Include also the totals down to see if no missing numbers.

Page 16: Table 4-Bi-variable and multivariable analysis for chorioamnionitis (n=356). There is no need of frequences unless it is the bivariate analysis with chi-square or Fisher test. Check if you can use them separately.

Page 16: Line 187: make a separate table showing the overall prevalence of chorioamnionitis and prevalence in different selected hospitals.

**Page 18: 203 Discussion**
Discuss your socio-demographic results.

**Conclusion:** Avoid discussion in conclusion, and conclude according to your findings

Page 21: 271 Ethical considerations: take this in methodology

**Contribution**: Put all authors contributions.

**References**

Just follow the Vancouver referencing style.
